# Double Pneumonia Complicated with Abortions

**Published:** 1892-02

**Authors:** William F. Waugh


### DOUBLE PNEUMONIA COMPLICATED WITH ABORTION.

BY WILLIAM F. WAUGH, M. D.

During the prevalence of influenza last winter, all my cases of pneumonia were treated by the use of hot poultices and the administration of the *Febricide Pills*. These pills contain quinine, acetanilide and *cocaine*. There is nothing secret about them, and the object of this note is not to claim superiority for any special maker of pills, but simply to advert to the merits of the formula. While the pills made by the *Health Restorative Company* were employed for convenience, and proved entirely satisfactory, any reliable retail druggist could prepare them quite as well except for the coating. But the combination is one of very great value, combining, as it does, an efficient antithermic with a cardiac tonic and a third drug quinine, which shares in the action of both the others and has a special effect in combating the tendency to suppuration. Perhaps in no disease do we meet with the indications for this combination so perfectly as in pneumonia. And in the pneumonia prevalent last winter we had an opportunity of putting our methods of treatment to the most severe test offered during the present generation.

My records show that during the grippe period I attended twenty-three cases of lobar pneumonia, all of which recovered. Ten of these were hospital cases; the remainder occurred in my private practice.

Out of this group I have selected the worst case as the one which was in the greatest danger of death. She was a stout German woman, illiterate, living in unsanitary quarters, with an inefficient nurse. Everything was against her; yet she recovered. She was about twenty-four years of age, of good constitution and habits, except that she used beer moderately but regularly. I have learned to look upon this as of bad omen in pneumonia. She had been much disturbed by her baby—about a year old—who had been sick for some time with the influenza. March 1st she was seized with a chill, and presented the characteristic symptoms of pneumonia. The disease rapidly extended to both lungs, until the dyspnœa was extreme. During the first three days my orders were not carried out by the drunken nurse, who was dismissed on March 3d. By this time the patient was in a pretty bad state, the respiration being carried on by the upper lobes alone; the lips blue; breathing very rapid; temperature running up to 104.5 deg. and pulse 136. Vomiting had been troublesome before her illness, as she was about five months pregnant. It was with difficulty we could get any food to remain in her stomach for a few minutes at a time. During the night of March 5th she aborted, losing but little blood—the fœtus coming away easily. By this time the nursing department had been reconstructed, and the food, medicine and poultices were attended to very nicely. Crisis occurred March 7th, and the case went on to recovery without any untoward incident. The patient was discharged from treatment on March 22d, convalescence being somewhat prolonged by the effects of the abortion, and by the bad hygienic surroundings. Alcoholic stimulants were used throughout in the same quantities the patient had been accustomed to before her illness. Febricide was the only drug given, except a few doses of wine of ergot, a teaspoonful each, just after the abortion. "The case was watched throughout with the utmost solicitude, and the unexpected recovery was looked upon by all who saw it, as a notable instance of the value of the combination employed."

Nor is the treatment of twenty-three cases without a death, during the prevalence of influenza, a record so common as to be undeserving of notice. The great work of Juergensen, in directing attention to the heart as almost the sole source of danger in pneumonia, has never received the general attention it deserves. People recover from pneumonia in spite of their treatment with antimony, veratrum, aconite, or the depressant antipyretics given alone, and that in all sufficient numbers to confirm their medical attendants in the use of these drugs.

But the magnificent results of Juergensen with hundreds of cases, and no deaths, except in the aged, have never been paralleled. His methods of cold baths, quinine, red wine and raw beef, seems too peculiar to find general acceptance in this country, where the cold bath has never really extended beyond hospital practice. The object, however, sought in my own method of treatment, is precisely the same as that of Juergensen—to sustain the heart, and by combating the fever that saps the strength of the heart and increases its labor. With him I say : It is the heart, always the heart. Here lies the danger ; here must be the remedy. Every means we employ for treatment acts for good only, as it strengthens this organ or relieves it of its burdens. Antipyretics accomplish the latter object. It is necessary to see that they do not hurt the patient more by lowering his cardiac power than they do good by lessening his fever.—*Times and Register.*

